# The molecular basis of cereal grain proteostasis

**DOI:** 10.1042/EBC20210041

**Published:** 2022-08-05

**Authors:** Hui Cao, Owen Duncan, A. Harvey Millar

**Affiliations:** 1 ARC Centre of Excellence in Plant Energy Biology and School of Molecular Science, The University of Western Australia, Bayliss Building M316, Crawley, WA 6009, Australia; 2 Western Australia Proteomics Facility, University of Western Australia, Bayliss Building M316, Crawley, WA 6009, Australia

**Keywords:** cereal grain development, proteases, proteostasis, storage protein

## Abstract

Storage proteins deposited in the endosperm of cereal grains are both a nitrogen reserve for seed germination and seedling growth and a primary protein source for human nutrition. Detailed surveys of the patterns of storage protein accumulation in cereal grains during grain development have been undertaken, but an in-depth understanding of the molecular mechanisms that regulate these patterns is still lacking. Accumulation of storage proteins in cereal grains involves a series of subcellular compartments, a set of energy-dependent events that compete with other cellular processes, and a balance of protein synthesis and protein degradation rates at different times during the developmental process. In this review, we focus on the importance of rates in cereal grain storage protein accumulation during grain development and outline the potential implications and applications of this information to accelerate modern agriculture breeding programmes and optimize energy use efficiency in proteostasis.

## Introduction

To meet the rising food demand from an increasing global population [1], agronomy, mechanisation, and adoption of advanced modern agriculture breeding technologies have significantly increased total grain yield over the past half century [2–4]. However, such improvements in grain yield can be accompanied by reduced grain quality. Central to this loss of quality is the well-known inverse relationship between grain yield and grain protein content [5,6]. Plant proteins represent 57% of total human dietary protein intake, and cereal proteins of maize, rice, and wheat account for a large portion of this total [7]. As grain proteins are key indices of cereal grain quality and market price, paying extra attention to grain protein content improvement and a better balance between grain yield and grain protein content are needed for more sustainable agriculture breeding programmes.

Cellular protein content in plants is constantly being replaced, so proteostasis is the combined result of both new protein synthesis rate and existing protein degradation rate. These processes are a substantial energy cost to cells as they involve ATP-dependent processes including protein biosynthesis, folding, trafficking, and degradation [8–10]. Biochemical evidence suggested that it requires 5.25 ATP molecules per amino acid molecule for amino acid synthesis, another 5.25 ATP per amino acid for polypeptide chain formation, a further 0.5–1.5 ATP per amino acid if resulting polypeptides need to be imported into cellular organelles, and 1.25 ATP per amino acid for the breakdown of polypeptides via the 26S proteasome [11–13]. While crop plants have access to a large source of ATP through photosynthesis, the majority of it is used to maintain fundamental biological functions and metabolic processes inside plastids. So, respiratory ATP from the degradation of photosynthetically derived sugars drives the accumulation and deposition of storage proteins in cereal grains during grain development [14,15]. Protein production is known to be energetically more expensive than starch synthesis on a mass basis [8,9,16], so the energy use efficiency of proteostasis (PEUE) is of particular importance in cereal crops as it will not only determine grain protein content but also impact on grain weight and thus yield.

## Storage proteins accumulation in cereal grains is a complicated and coordinated process

Storage proteins are the second most abundant macromolecules after starch in mature cereal grains, accounting for 8–18% of grain on a dry weight basis. These proteins rapidly accumulate during the grain filling stage of grain development, and the nitrogen reserves present in them are critical for future grain germination and early seedling development [17–20]. Cereal grain storage proteins can be experimentally fractionated into four groups according to their solubility: namely albumins (soluble in water), globulins (soluble in dilute salt), prolamins (soluble in aqueous alcohol, such as 70% ethanol and 55% propanol), and glutelins (soluble in dilute acid or alkali solutions) [21]. Wheat dough quality and bread-making quality are tightly linked with total grain protein content, the ratio of particular gluten monomer (glutenin-to-gliadin ratio), and the ratio of high-molecular glutenin subunit (HMW-GS) to low-molecular glutenin subunit (LMW-GS) within the group of glutenin [22–24].

**Figure 1:**
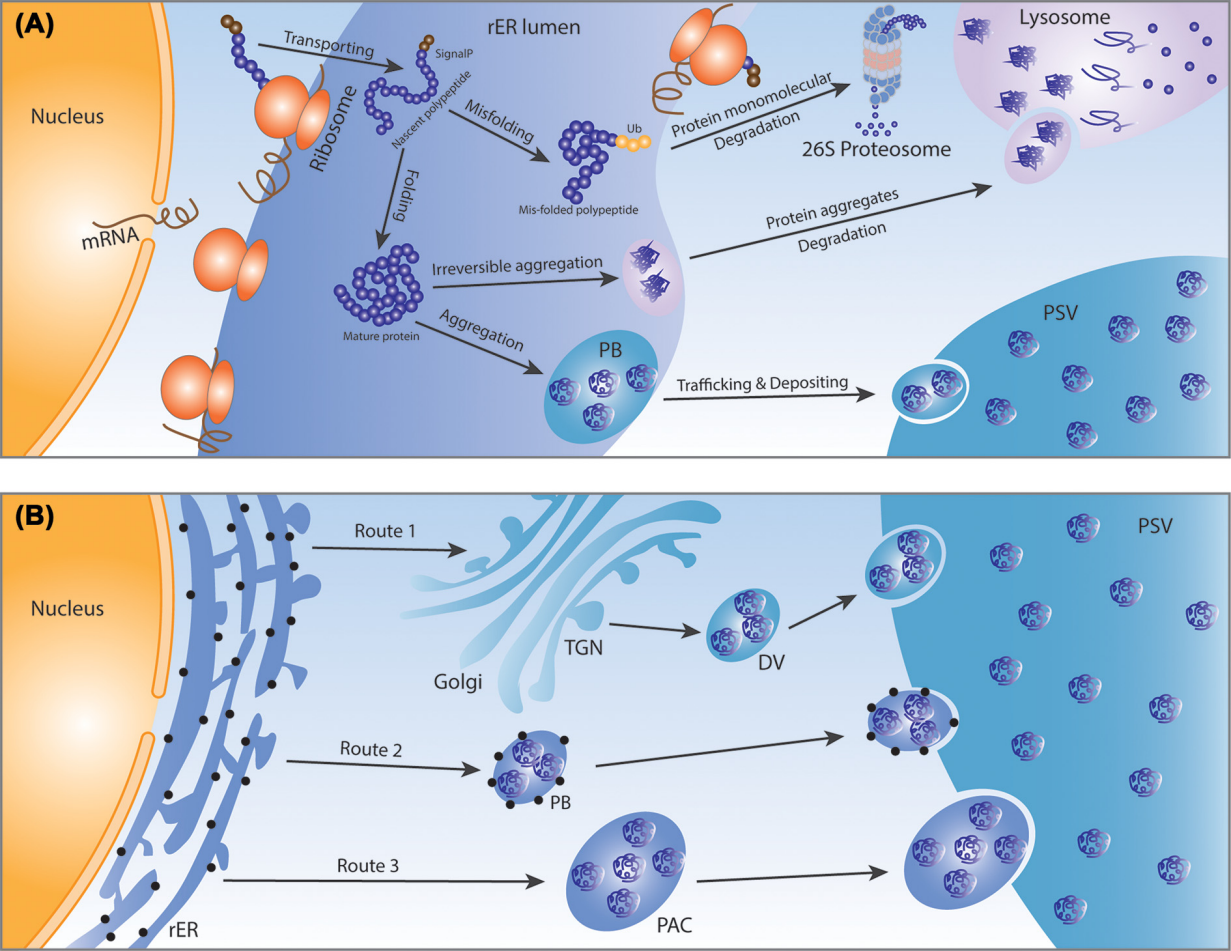
Cereal grain synthesis, degradation, and deposition of storage proteins (**A**) The synthesis and degradation of cereal grain storage proteins as typically observed in maize, rice, and wheat. Storage proteins are generally synthesized on the rER and then translocated into the rER lumen by N-terminal signal peptides. Once signal peptides are cleaved off, a maturation process transforms nascent polypeptides into fully mature, stable, and storage proteins. Major events that participate in the maturation process include polypeptide folding [46,78], inter- and intra-disulfide bond formation [25,79–81], post-translational modifications [82–85], and protein sorting [28,32,86]. Mis-folded polypeptides are tagged by ubiquitin ligases and delivered to the 26S proteasome of the UPS for degradation and subsequent amino acid recycling. Properly folded mature protein aggregates are temporarily placed in PB before being transported into PSV for permanent storage, while irreversible aggregates are delivered to lysosomes for degradation and amino acid recycling [46,87]. (**B**) The major trafficking routes of storage proteins in cereal grains. There are three main trafficking routes in cereal grains to allow transport of storage proteins from rER to PSV [36]. The classic *route 1*, also known as the Golgi-dependent route, involves mature storage proteins, such as albumins and globulins, being transported into the Golgi, followed by the formation of the DV in the TGN and the merger of DV into the PSV. Prolamin aggregates are often deposited via *route 2* and *3* that are categorized as Golgi-independent routes [29]. In *route 2*, ER-derived protein bodies containing storage protein aggregates are formed, which are then delivered to and fused with the PSV. Some storage proteins are deposited through *route 3*, in which protein aggregates are firstly gathered in the ER-derived PCA and then directly delivered into the PSV. Protein aggregates containing temporary protein vesicles like DV, PB, and PAC are released into the PSV via an autophagy-like process. Only the commonly shared mechanisms among major cereal crops are presented to give an overview of the process. Further detail of the sorting and trafficking mechanisms and the discussion of differences in these processes between cereal crop species are reviewed elsewhere [33–36]. Abbreviations: DV, dense vesicle; PB, protein body; PAC, precursor-accumulating vesicles; PSV, protein storage vacuole; rER, rough endoplasmic reticulum; SignalP, signal peptide; TGN, trans-Golgi network; Ub, ubiquitin ligases.

Like most secretory proteins, cereal grain storage proteins are synthesized on polyribosomes that are situated on the rough endoplasmic reticulum (rER). After locating to the rER lumen, the newly synthesized polypeptides are guided through a highly coordinated process to become mature proteins. In this process, proper structural folding and protein aggregation are regulated by chaperones, protein disulfide isomerase (PDI) and various post-translational modifications (PTM) (Figure 1A). Quantitative proteomics data obtained from wheat grains indicate that the abundance of PDI family isoforms increased 4-fold in 10 days from 7 to 17 days post anthesis (DPA) [14] followed by a decline during the grain maturation stage [25]. Misfolded proteins and irreversible protein aggregates are degraded by either the ubiquitin–proteasome system (UPS) or the autophagy-lysosome system (Figure 1A). Once folding and aggregation are completed, mature storage proteins and aggregates are transported into protein storage vacuoles (PSV) in grain endosperm cells through three distinct secretory processes (Figure 1B). The trafficking process starts with the sorting of cargo proteins into storage vacuoles. Vacuolar sorting receptors (VSRs) are the key participants in vacuolar protein sorting, they recognize various types of vacuolar sorting determinants (VSDs) in storage proteins via protein–protein interactions [26,27]. The range of vacuolar protein sorting mechanisms and the essential roles of VSRs in storage proteins sorting has been intensively characterized in different crops [28–31]. Recent quantitative data suggest that the protein abundance of VSRs and/or the expression of the genes that encode them are increased by up to 3-fold in cereal grains during grain development to facilitate the high demands of storage protein sorting and trafficking [14,32]. Further detail of the sorting and trafficking mechanisms of storage proteins in cereals as well as differences in these processes between cereal crops are reviewed elsewhere [33–36].

## Protein turnover occurs during all stages of cereal grain development

Significant interest has been paid to understanding the role of protein synthesis and degradation during protein accumulation in cereal grains. The majority of this work is based on measuring changing patterns of protein abundance in time course proteomics experiments, and matching this to related changes in transcriptomics and metabolomics experiments [37–43]. Proteins that are required for cell division, cell wall formation, and cell growth, such as cytoskeleton proteins, histones, and xyloglucan endotransglucosylases/hydrolases accumulate in the early stage of grain development [42,44,45]. During the grain filling stage, the accumulation of these cell growth/division related proteins stops and the degradation of them begins. The accumulation of grain storage proteins, enzymes for starch synthesis, protein synthesis machinery, and factors for protein folding and post-translational modification of storage proteins increases dramatically during the short period that begins from grain filling [14,39,45]. Once grain starch and storage proteins reached their maximum abundance, the grain maturation stage begins during which ubiquitin is accumulated and activated to facilitate the recycling of unwanted proteins. For example, enzymes of starch synthesis and those required for storage protein synthesis decrease in abundance during maturation. A large number of stress response proteins, such as heat shock proteins (HSPs) and late embryogenesis abundant (LEA) proteins, accumulate during the grain maturation stage [42,46,47]. From experimental evidence in non-plant systems [48,49], LEA proteins are believed to have important functions in stabilizing proteins and preventing functional proteins from unwanted aggregation. Cell biology processes also contribute to maturation, notably programmed cell death in the endosperm [50]. Detailed discussion of the impact of programmed cell death in wheat [51], barley [52], and maize [53] seed maturation processes are available elsewhere. Our recent development and use of ^15^N labeling of wheat plants post-anthesis to measure precise rates of synthesis and degradation of specific proteins during wheat grain development provide another window into protein turnover. Rates are only available for proteins with high enough abundance to be identified by mass spectrometry and synthesis rates that allow detection and quantitation of both old and new protein populations at the same time in a given sample. Currently, this work has been done in hydroponic culture and advancements are needed to deploy it in the field to observed rates in natural conditions [14].

## Protein synthesis during accumulation of proteins in cereals

It is clear from available evidence that protein synthesis is a primary driver of changes in protein abundance during grain filling in cereals. In maize, over 90% of the storage proteins accumulate during the grain filling stage from 8 to 34 DPA. Zeins, the most abundant storage proteins in maize, rapidly and steadily accumulate in abundance across the entire grain filling stage, and the abundance of globulins and vicilin-like proteins increase more than 10-fold from 15 and 30 DPA [39,54]. During the same time period, the abundance of carbohydrate metabolism-related proteins increase from 15 to 25 DPA, followed by a decrease from 30 DPA onwards [39]. Similar results are observed in rice grains where starch and protein synthesis-related proteins accumulate in abundance from 6 to 20 DPA followed by an accumulation of starch and storage proteins [45,47]. In wheat grains, apart from a few exceptions where HMW-GS and gliadins are reported to accumulate synchronously and are co-located in the central endosperm [55,56], the accumulation of gliadins occurs at early- and mid-grain filling stage from 6 to 20 DPA, while other storage proteins like globulins, avenin-like proteins and glutenin subunits tend to accumulate at mid- and late-grain filling stages; 14–28 DPA [37,44]. The abundance of wheat grain storage proteins can increase more than 25-fold on average and specific proteins up to 126-fold (e.g. globulin 1) during the 10 days from 7 to 17 DPA, suggesting very rapid protein synthesis rates during grain filling in wheat [14]. Combining *in vivo* stable isotope labelling and in-depth quantitative proteomics has shown that the median relative protein synthesis rate for wheat grain storage proteins was approximately 200% per day, with particular proteins exceeding a relative rate of 500% per day [14].

The biosynthesis of non-storage proteins in cereal grains at different stages of grain development boosts nutrition reserve deposition in forms that can be rapidly mobilised during germination. To be ready for the deposition of starch and storage proteins during the grain filling stage, cereal grains in the pre-grain filling stage produce more cells through cell division and loosen and expand cell walls making space available in each cell. Yu et al. reported that cell wall formation-related enzymes, such as UDP-glucose 6-dehydrogenases and xyloglucan endotransglucosylases/hydrolases, accumulate in maize at the early grain development stage to form cell membranes and extend cell walls [42]. Increased abundance of starch synthesis enzymes: e.g. sucrose synthase (SuSy, 6.3-fold), ADP-glucose pyrophosphorylase (AGPase, 7.5-fold), 1,4-alpha-glucan-branching enzyme (BE, 8.3-fold); and protein synthesis enzymes, ribosomal large subunits (2.4-fold) and ribosomal small subunits (2.5-fold) were observed in wheat from 7 to 17 DPA [14].

PDIs are known to participate in the formation of intermolecular or intramolecular disulphide bonds for the assembly of glutenin macropolymers. The abundance accumulation of five different groups of PDIs are boosted during grain filling in wheat [25]. A primary event during grain maturation in cereals is the prevention of irreversible aggregation of storage proteins following loss of moisture during grain desiccation [48,49,57,58]. Chaperones, HSP, and LEA proteins have been proposed to have roles in preventing aggregation and accumulate during late grain development stage in major cereal crops [37,42,47].

## Protein degradation is important for cellular proteostasis maintenance and storage proteins accumulation

Protein degradation occurs outside the grain to enable nitrogen remobilization to grains from flag leaves, stems, and roots through processes of plant senescence. Senescence is a programmed degradation of cell constituents that makes nutrients available for grain filling through the breakdown of chloroplast machinery and the catabolism of Rubisco, chlorophyll, and macromolecules mediated by UPS or the autophagy-lysosome system [59]. The accumulation of organic nitrogen-rich compounds before anthesis is a major source of grain nitrogen in cereal crops [60–62]. The role of protein degradation inside grains during their development is more rarely investigated [63,64]. However, this process is needed to recycle unwanted and dysfunctional cytosolic proteins within the grain, for both cellular proteostasis maintenance and nitrogen recycling of storage proteins. Rapid, triggered degradation of grain cell growth/division proteins at the grain filling stage and starch synthesis enzymes and ribosomes at the grain maturation stage is commonly observed in cereal crops [39,44,45]. Our proteomics comparison of high protein wheat lines expressing an extra gene for HMW glutenin (Ay HMW-GS) is consistent with the ubiquitin–proteasome system recycling nutrients from unwanted proteins, providing a positive reinforcement and resources for more storage protein synthesis and deposition [46].

**Figure 2:**
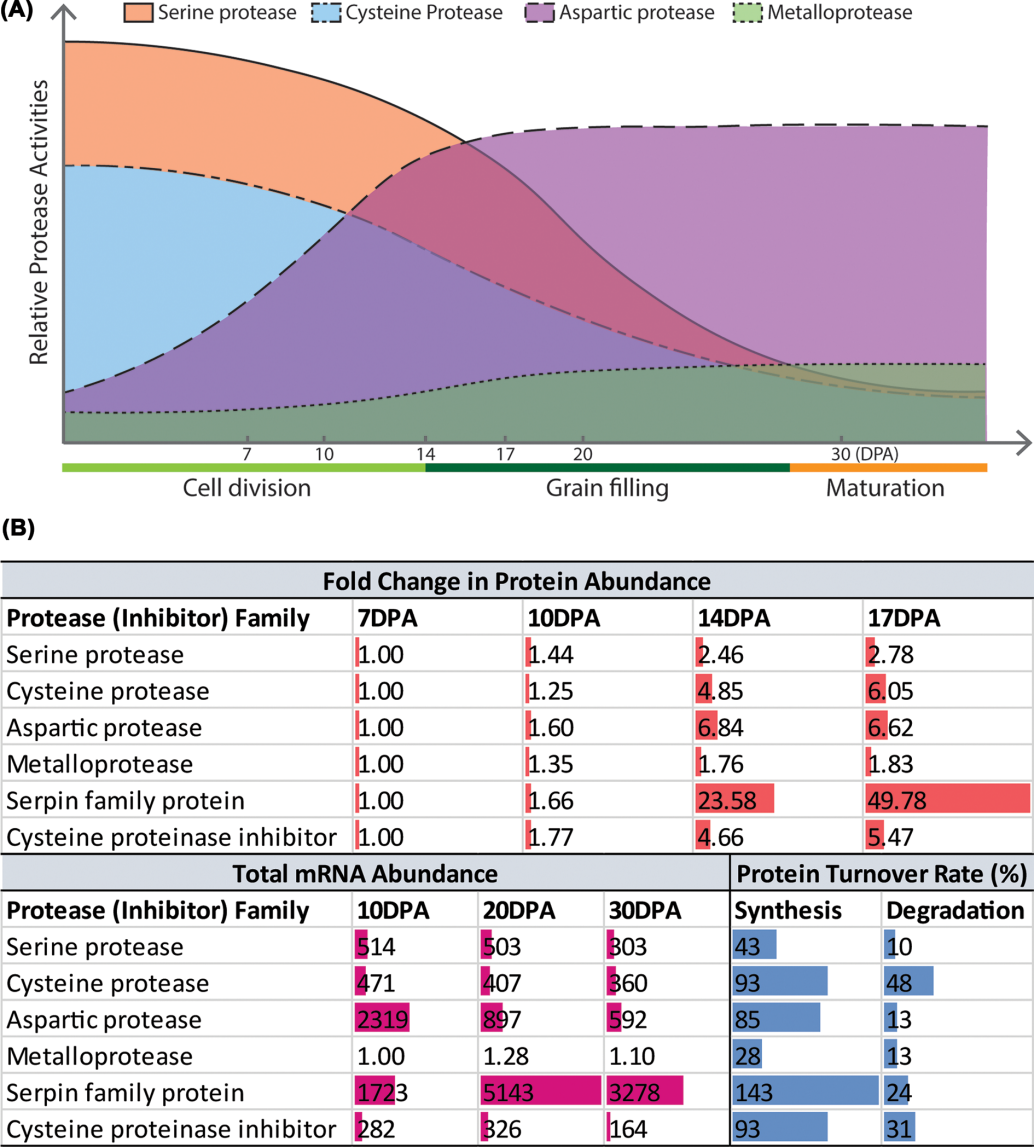
The accumulation profile of the four major protease and two major protease inhibitor classes in wheat grains during grain development (**A**) The relative protease activities of four major protease families in wheat grain during grain development. Relative protease activities were summarized from previously reported enzyme assays [65] and are broadly consistent with recent quantitative proteome data [14]. (**B**) The fold change in protein abundance, total mRNA abundance (transcripts per million) and protein synthesis and degradation rates (in % change per day) of the four major protease families and the two protease inhibitor families during wheat grain development. The fold change in protein abundance and protein turnover rate data were originally reported by Cao et al. in 2022 [14], while the transcript data were originally reported by Pfeifer et al. in 2014 [67]. The averaged fold change in protein and the total mRNA abundance are presented for each family of proteins or transcripts. The median protein synthesis and degradation rates of each protein family during early- and middle grain filling stages from 7 to 17 DPA are also presented.

Four major protease families are detectable in cereal grains during grain development, namely serine proteases, cysteine proteases, aspartic proteases, and metalloproteases [65,66]. Enzyme assay reveals that total endoproteolytic activity is detectable in the early stages of grain development, and quickly reaches a maximum level that is maintained until the end of grain maturation [65]. Proteolytic activities by serine and cysteine proteases peak at early stages of grain development and their levels decrease at intermediate and late stages. The activities of aspartic proteases and metalloproteases are detectable at intermediate grain filling stages but are relatively higher at later stages of grain development (Figure 2A). In wheat, the abundance of proteins in all four protease families increased during the grain growth period from 7 to 17 DPA, while the transcripts encoding them decreased over time from 10 to 30 DPA (Figure 2B). A significant accumulation of serpin and cysteine protease inhibitors (50- and 5.5-fold on average over 10 days from 7 to 17 DPA) was observed in quantitative proteome data and the genes that encode them are induced in transcriptome studies [14,67]. These inhibitors will suppress the activity of serine proteases and cysteine proteases at the late grain development stage, and slow the degradation of storage proteins before germination (Figure 2B). Measurements of protein turnover rates in developing wheat grains have revealed that these proteases and protease inhibitors are relatively stable proteins that have higher protein synthesis rates and similar degradation rates than the average rates of all proteins in grains (median synthesis rate of 35% per day and median degradation rate of 11% per day) (Figure 2B) [14]. More detail on the involvement of proteases in development and germination of cereal grains is also reviewed elsewhere [66,68], as is the role of cell biology processes such as PCD [50].

Cereal grain storage proteins are typically considered stable proteins, at least during grain filling and maturation, and are mostly treated as protein groups that are made and stored in the grain endosperm without appreciable turnover [69,70]. However, this is somewhat at odds with the fact that these proteins are evolutionarily optimised for rapid degradation as nitrogen sources during grain germination and early seedling growth. Our recent study following the dynamic rate of protein synthesis and degradation has revealed that wheat grain storage proteins are less stable than previously thought during grain development. They have considerably higher degradation rates (*K*_D_) than the average for all grain proteins [14]. Quantitative data suggested that storage proteins belong to the most unstable protein group among all protein groups in the wheat grain during the grain filling stage. The median relative degradation rate of storage proteins is 48% per day (i.e. a half-life of <1.5 days), which is over 4 times faster than the overall degradation rate of grain proteins (median *K*_D_ of 11% per day, i.e. half-life of >6 days) and 16 times faster than stable photosynthesis related enzymes like photosystem II reaction centre protein H (median *K*_D_ of 3% per day, i.e. a half-life of >23 days) [14].

## Protein turnover ATP energy cost distribution in wheat grains during grain filling

The fact that storage proteins undergo substantial rounds of synthesis and degradation (albeit alongside net protein accumulation during grain filling) might just be a biological oddity of no particular significance if it was not for the substantial ATP demand it places on polypeptide chain formation in the grain. The majority of ATP production in cereal grains comes from mitochondrial oxidative phosphorylation that comes at the expense of sugar catabolism that might otherwise be substrates for starch synthesis [71]. As a consequence, this process impacts substantially on the ATP budget for nutrient reserves deposition and other biological events during grain development [72]. Studies of energy budgets for protein turnover and their implications are still at an early stage, especially in cereal crops. Several attempts have been made recently to understand the implications of these ATP costs, such as the energy cost for maintaining proteostasis of different subcellular structures and metabolic pathways in *Arabidopsis* rosettes and changes in them during the diurnal cycle and in response to high light [12,73,74]. Our recent study on the ATP energy budget for protein turnover and cellular proteostasis maintenance in wheat grains during grain filling, also provides an initial quantitative understanding of how different wheat grain proteins accumulate and calculates the energy costs for their accumulation during grain development [14]. Data for 1140 grain proteins indicated that wheat grains invested 18% of total ATP production generated from oxidative phosphorylation into protein synthesis and 2% into protein degradation. Nearly half of the energy budget of total protein synthesis and a quarter of the total energy budget for protein degradation was used for synthesis and degradation of storage proteins and enzymes of major carbohydrate metabolism. This analysis also revealed that wheat grain storage proteins were the most expensive protein functional category in terms of synthesis or maintenance of proteins. We calculated that approximately 25% of the newly synthesized storage proteins are degraded before they make it to storage vacuoles [14]. Taken together, this evidence suggests that wheat grains have a relatively low PEUE despite their rapid storage protein production rates.

## Conclusion and future perspectives

**Figure 3:**
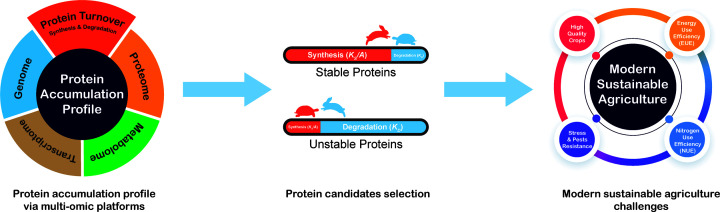
Role of protein turnover analysis in sustainable agricultural advancement through analysis of protein accumulation profiles The abundance of proteins are directly determined by the relative rates of their synthesis and degradation. Adopting breeding strategies that include optimizing energy use efficiency of protein production (PEUE) in crop plants provides a valuable focus for breeding high quality crops in a more sustainable way. Targeting proteins with a specific stability status, selected from protein turnover data and/or protein accumulation profiles integrated by multi-omics data, could enhance PEUE and can be altered using either conventional cross breeding approaches or advanced gene editing technologies.

The measurement of protein synthesis rate and protein degradation rate in cereals [14] offers a pathway to look beneath the poor correlation between the transcriptome and proteome in plants [75–77] to enable the direct study of protein synthesis, storage and degradation over time. This approach can quantify the most stable proteins and the least stable proteins *in vivo*, enabling the selection of candidate proteins for multiple purposes in cereal breeding programmes (Figure 3). Breeding strategies that reduce the turnover of storage proteins during grain filling (e.g., by knocking out unstable storage proteins and expressing genes for more stable versions or decreasing the abundance or targets of proteolytic machinery) could help optimize PEUE, increase grain protein content and improve grain quality in crops. This strategy aims to increase storage protein deposition by reducing the turnover of existing storage proteins, rather than demanding more ATP from energy budgets for making new proteins. This approach has the potential to contribute to alleviating the inverse relationship between grain yield and grain protein content that is commonly observed in crops. NUE and EUE are commonly considered independently in plants; however, in PEUE, they are united by considering the energetic cost of maintaining N within the polypeptide pool in plant tissues. Improvement in crop NUE could also benefit from lowering the cost of protein turnover and returning the budget for use in N assimilation and transportation.

## Summary

- Protein synthesis and degradation rates underpin cellular proteostasis and their imbalance is needed for protein accumulation.
- Wheat grain storage proteins have significantly higher degradation rates than the average of all grain proteins, implying that storage proteins are more unstable during grain filling than previously thought.
- Breeding strategies involving knocking out unstable storage proteins, expression of genes for more stable versions, or removing proteolytic machinery, may influence the inverse relationship between grain yield and grain protein content in crops.

## Open Access

Open access for this article was enabled by the participation of The University of Western Australia in an all-inclusive *Read & Publish* agreement with Portland Press and the Biochemical Society under a transformative agreement with CAUL.

## Abbreviations

DV: dense vesicle
HMW-GS: high-molecular glutenin subunit
LMW-GS: low-molecular glutenin subunit
PAC: precursor-accumulating vesicle
PB: protein body
PDI: protein disulfide isomerase
PSV: protein storage vacuole
rER: rough endoplasmic reticulum
SignalP: signal peptide
TGN: trans-Golgi network
Ub: ubiquitin ligases
UPS: ubiquitin–proteasome system
VSD: vacuolar sorting determinant
VSR: vacuolar sorting receptor
